# High-Bandwidth Heterodyne Laser Interferometer for the Measurement of High-Intensity Focused Ultrasound Pressure

**DOI:** 10.3390/mi14122225

**Published:** 2023-12-11

**Authors:** Ke Wang, Guangzhen Xing, Ping Yang, Min Wang, Zheng Wang, Qi Tian

**Affiliations:** 1 Division of Mechanics and Acoustics, National Institute of Metrology, Beijing 100029, China; wangke@nim.ac.cn (K.W.); xinggz@nim.ac.cn (G.X.); wangm@nim.ac.cn (M.W.); tianqi@nim.ac.cn (Q.T.); 2 Ultra-Precision Optoelectronic Instrument Engineering Center, School of Instrument Science and Engineering, Harbin Institute of Technology, Harbin 150080, China; 18b901009@stu.hit.edu.cn

**Keywords:** high-bandwidth heterodyne laser interferometer, sound pressure measurement, high-intensity focused ultrasound, optical carrier frequency

## Abstract

As a high-end medical technology, high-intensity focused ultrasound (HIFU) is widely used in cancer treatment and ultrasonic lithotripsy technology. The acoustic output level and safety of ultrasound treatments are closely related to the accuracy of sound pressure measurements. Heterodyne laser interferometry is applied to the measurement of ultrasonic pressure owing to its characteristics of non-contact, high precision, and traceability. However, the upper limit of sound pressure measurement is limited by the bandwidth of the interferometer. In this paper, a high-bandwidth heterodyne laser interferometer for the measurement of high-intensity focused ultrasound pressure is developed and tested. The optical carrier with a frequency shift of 358 MHz is realized by means of an acousto-optic modulator. The selected electrical devices ensure that the electrical bandwidth can reach 1.5 GHz. The laser source adopts an iodine frequency-stabilized semiconductor laser with high-frequency spectral purity, which can reduce the influence of spectral purity on the bandwidth to a negligible level. The interference light path is integrated and encapsulated to improve the stability in use. An HIFU sound pressure measurement experiment is carried out, and the upper limit of the sound pressure measurement is obviously improved.

## 1. Introduction

The calibration of sound pressure is very important to ensure the safety of HIFU treatment [1,2,3]. Because of the advantages related to its non-contact nature, high precision, high bandwidth, and direct traceability to the laser wavelength [4,5], heterodyne laser interferometry is adopted by the new generation of sound pressure reference [6,7,8]. The maximum sound pressure for HIFU treatment can reach 60 MPa or even higher [9]. However, the maximum value of an HIFU sound pressure measurement based on laser interferometry can be seen in the current literature is around 10 MPa [10]. The upper limit of a sound pressure measurement seriously lags behind the application of HIFU treatment technology. One of the main reasons for this limitation is the insufficient bandwidth of the heterodyne laser interference system, including the optical bandwidth and the electrical bandwidth. The optical bandwidth is mainly dependent on the optical carrier frequency of the heterodyne interferometer and should be able to cover the optical Doppler frequency shift, which is proportional to the measured vibration velocity. The electrical bandwidth is decided by the bandwidth of the photodetector and subsequent signal-processing circuits. Also, the electrical bandwidth should correspond to the bandwidth of the interference signal output by the photodetector.

The bandwidth of a heterodyne interferometer directly determines the upper limit of a sound pressure measurement. Through theoretical analysis, Daniel Royer identified that the bandwidth of the interference system changes linearly with the sound pressure [11]. According to this research, to realize a measurement with a frequency of 1 MHz and sound pressure of 60 MPa, the bandwidth should be greater than 130 MHz. Our study is based on the condition that the sound field is linear. However, under high sound pressure, the HIFU sound field exhibits severe nonlinearity, causing the calculation of system bandwidth to deviate from the actual situation. In our previous research, it was demonstrated through a combination of experiments and simulations that the bandwidth of the laser interference system should change in a quadratic manner with the HIFU sound pressure [12]. Therefore, when conducting an HIFU sound pressure measurement, as the sound pressure increases, the demand for the bandwidth of the laser interference system will sharply increase.

To realize an HIFU sound pressure measurement, a high-bandwidth heterodyne laser interferometer is urgently needed. There are currently several optical carrier acquisition methods based on the Zeeman effect [13,14], stress-induced birefringence [15,16,17], and acousto-optic modulators [18,19]. Given that most methods are designed for displacement measurements, their demand for optical carriers generally does not exceed tens of megahertz, and the laser source is typically a He-Ne laser, which has better frequency stability. Meanwhile, in the measurement of HIFU sound pressure, optical carriers in the order of hundreds of megahertz are needed.

An acousto-optic modulator (AOM) uses the acousto-optic interaction to obtain the frequency shift of light. When a laser is diffracted by ultrasonic grating through an acousto-optic medium, its propagation direction and frequency will change. The frequency of the first-order diffracted light superimposes an ultrasonic frequency on the original input laser frequency, and the frequency shift range can reach from tens of megahertz to hundreds of megahertz. Therefore, an AOM is suitable for a laser heterodyne interference system with a large frequency difference.

In this paper, a high-bandwidth laser heterodyne interferometer based on an AOM for a hundred-megapascal-level HIFU sound pressure measurement is developed and tested. The selected AOM can perform an optical frequency shift of about 358 MHz. The influence of the spectrum characteristics of the laser light source on the bandwidth of the interference system is analyzed, and the interference signal spectrum is tested using two kinds of light sources. The optical path is designed, and finally, the system is integrated and encapsulated to ensure the stability of the system.

## 2. System Design

### 2.1. Principle of HIFU Sound Pressure Measurement Based on Laser Interferometry

**Figure 1:**
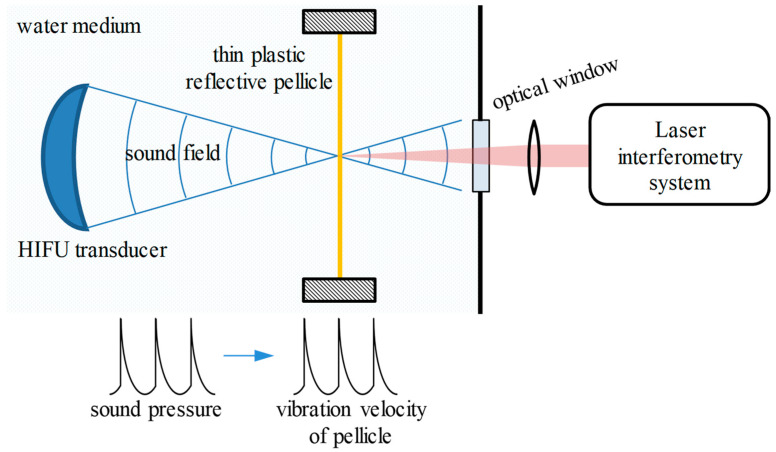
Schematic diagram of the HIFU sound pressure measurement based on laser interferometry. The blue line represents the sound field, the yellow line represents the thin plastic reflective pellicle, and the red line represents the laser.

The schematic diagram of the HIFU sound pressure measurement based on laser interferometry is shown in Figure 1. The HIFU transducer and thin plastic reflective pellicle are placed in the water tank. The sound axis direction is perpendicular to the pellicle, and the sound focal point is located on the pellicle. In order to ensure that the vibration of the pellicle follows the sound wave, the thickness of the pellicle needs to be much less than the wavelength of the sound wave. The thickness of the pellicle used in the experiment is 15 μm. The wavelengths of the acoustic waves corresponding to the frequencies of 1 MHz and 3 MHz are 1.5 mm and 0.5 mm, respectively, which are far greater than the thickness of the pellicle.

The measuring beam of the laser heterodyne interference system measures the vibration velocity of the pellicle through the optical window, and the sound pressure can be measured after signal processing. The relationship between the sound pressure *p*(*t*) and the vibration velocity *v*(*t*) can be expressed as follows:

*p*(*t*) = *ρcv*(*t*), (1)

where *ρ* represents the density of the water medium and *c* represents the sound velocity in the water medium [20].

As the sound pressure increases, the HIFU sound field transforms into a nonlinear sound field, meaning that the sound pressure waveform is no longer in the form of cosines, but appears in a form with rich multiple harmonics, as shown in Figure 1. This is also the main reason for the rapidly increasing demand for the bandwidth of laser interference systems in the HIFU sound pressure measurement.

### 2.2. Optical Path Design

According to the principle of laser interference, the measuring beam and the reference beam of the laser heterodyne interference system can be expressed as follows:

(2) $$E_{m}=A_{m}e^{-i\left[2\pi f_{m}t+\frac{4\pi nx(t)}{\lambda }\right]},$$

(3) $$E_{r}=A_{r}e^{-i2\pi f_{r}t},$$

where *A*_m_ and *A*_r_ represent the amplitude of the laser beam, *f*_m_ and *f*_r_ represent the laser frequency, *n* represents the refractive index of the medium, *x*(*t*) represents the displacement of the pellicle over time, and *λ* represents the vacuum wavelength of the laser. The 2nx(*t*)/*λ* part in Formula (2) represents the optical frequency change introduced by the measured vibration, which is the Doppler frequency shift *f*_d_. Therefore, the relationship between the Doppler frequency shift of the measuring beam *f*_d_ and the velocity of the measured object can be expressed as follows:

(4) $$f_{d}(t)=\frac{2n}{\lambda }v(t),$$

Combining Formula (1) and Formula (4), the relationship between the Doppler frequency shift and the measured sound pressure can be expressed as follows:

(5) $$f_{d}(t)=\frac{2n}{\lambda }\frac{p(t)}{\rho c},$$

Due to limitations such as photoelectric detection and signal demodulation, the optical carrier frequency of the laser heterodyne interferometer *f*_c_ must be greater than the Doppler frequency shift. Based on the above analysis, it can be concluded that the demand for the optical bandwidth varies linearly with the measured sound pressure. In order to achieve the measurement of the HIFU sound pressure with a maximum value of 100 MPa, the optical carrier frequency of the laser heterodyne interference system should be greater than 285 MHz (*n* = 1.333, *λ* = 633 nm, *ρ* = 1000 kg/m^3^, *c* = 1480 m/s).

**Figure 2:**
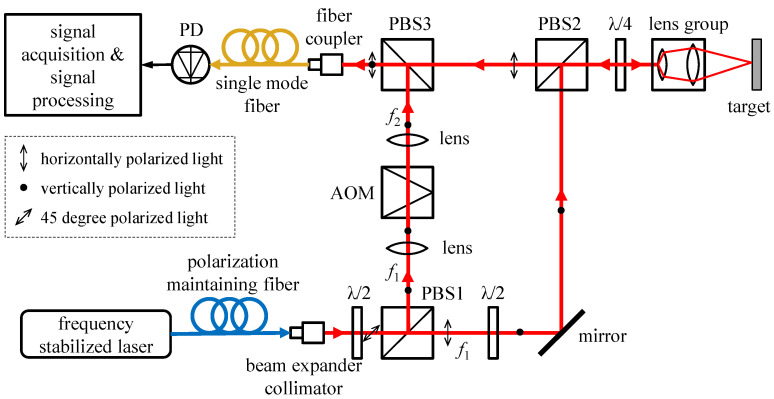
Schematic diagram of the optical path of the high-bandwidth laser heterodyne interference system.

To realize the above-mentioned purpose, this article uses an AOM to obtain a high-frequency optical carrier, and the optical path of the designed high-bandwidth laser heterodyne interference system is shown in Figure 2. The laser with frequency *f*_1_ emitted by the frequency-stabilized laser is emitted after passing through the polarization-maintaining fiber and the beam-expanding collimator. After passing through the λ/2 wave plate, the laser is divided into two by PBS1 (polarized beam splitter). The transmitted light, as a measuring beam, passes through the λ/2 wave plate, reflector, PBS2, λ/4 wave plate, and lens group and is incident on the surface of the measured object. The measuring beam reflected by the target passes through the λ/4 wave plate again, is transmitted at PBS2, and is incident on the optical fiber coupler after passing through PBS3. The reflected beam of PBS1 passes through the lens and AOM, and its frequency changes from *f*_1_ to *f*_2_. This beam is used as a reference beam and is incident on the optical fiber coupler after being reflected by PBS3. The reference beam and the measuring beam interfere at the fiber coupler and are coupled with the single-mode fiber. Through the interference signal formed by the photodetector (PD), the measured vibration information is obtained through signal processing.

**Figure 3:**
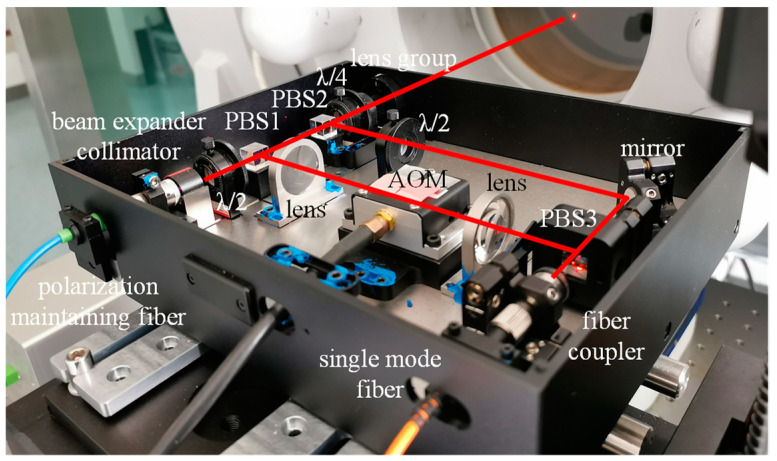
Integrated interference system.

The AOM is produced by AA Sa Opto-Electronic, and its model ID is MT350-A0.12. Its frequency shift size is about 358 MHz. Since the effective optical aperture of the AOM is only 0.12 mm, in order to improve the light energy utilization ratio, two convergent lenses are used before and after the AOM, and the focus of the two lenses is at the center of the effective working area of the AOM. To improve the spatial resolution of the sound pressure measurement, a variable focal length lens group was designed at the output end of the measuring light. By adjusting the focal length, the optical focus coincides with the position of the thin film. To improve the stability and convenience of the interference system, the light source and photodetector are connected to the optical path by an optical fiber. The interference system is integrated, as shown in Figure 3.

### 2.3. Electrical Signal Processing Section

According to our previous research [12], a relationship between the peak value of the measured HIFU sound pressure and the demand for electrical bandwidth has been established. Interference signals under nonlinear sound field conditions can be represented as follows:

(6) $$I(t)=Acos\left[2\pi f_{c}t+\frac{4\pi }{\lambda }\sum_{i=1}^{n}a_{i}\cos(2\pi f_{i}t+\varphi_{i})\right],$$

where *a_i_*, *f_i_*, and *φ_i_* represent the amplitude, frequency, and phase of each harmonic frequency component in the HIFU sound field. Due to the inability of this expression to perform function expansion, a combination of measured data and numerical simulation was used to analyze its bandwidth.

**Figure 4:**
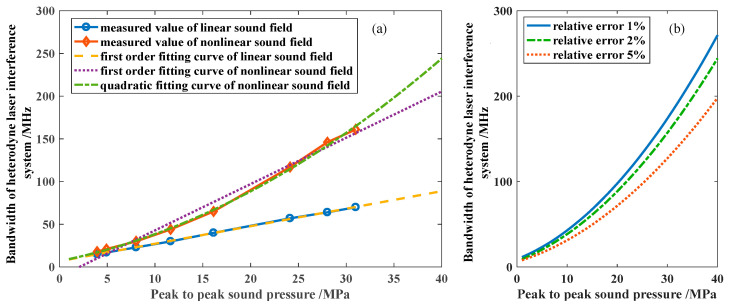
Electrical bandwidth of the heterodyne interference system versus the peak value of the measured sound pressure. (**a**) When the relative error of the sound pressure is less than 2%; (**b**) quadratic fitting curves with different relative error requirements under nonlinear sound field.

By using a high-intensity hydrophone to measure the sound pressure curves under different sound pressures, the corresponding ideal laser heterodyne interference signals can be calculated. By conducting numerical simulation analysis on the interference signal, its spectral distribution is obtained. In the case of limited bandwidth, the corresponding relationship between the measured peak sound pressure and the electrical bandwidth requirement of the laser heterodyne interference system under a certain relative sound pressure measurement error is obtained through numerical simulation. The trend of changes in different sound pressure and bandwidth requirements was fitted as shown in Figure 4, and the relationship between the electrical bandwidth requirements and peak sound pressure was obtained as a quadratic law.

**Figure 5:**
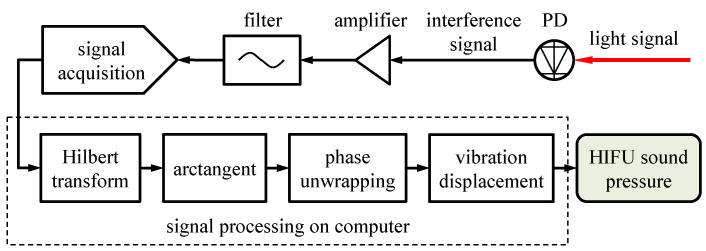
Schematic diagram of the electrical signal processing part of the proposed interferometer.

When the relative error is less than 2%, the relationship between the electrical bandwidth of the laser heterodyne interference system *B* and the peak sound pressure obtained through fitting can be expressed as follows:

(7) $$B=0.0924p^{2}+2.23p+6.93,$$

where *p* represents the peak sound pressure. Based on the above research, the electrical bandwidth needs to reach approximately 1.15 GHz to meet the sound pressure measurement target of 100 MPa. The electrical signal processing part of the proposed high-bandwidth interferometer is shown in Figure 5.

The interference signal output by the PD is amplified and filtered and then collected into the signal processing software on a computer through the signal acquisition unit. In the upper computer, the interference signal undergoes a Hilbert transformation, arctangent processing, and phase unwrapping to obtain vibration displacement information, and finally, the HIFU sound pressure is calculated. The selected PD is the FPD310-FC-VIS high-sensitivity fast PIN photodetector from Menlo Systems, with a bandwidth of 1.5 GHz. The signal acquisition device uses Teledyne LeCroy’s WAVERUNNER 9404 M digital oscilloscope, with a bandwidth of 4 GHz. The bandwidth of the devices used in the electrical signal-processing process can meet the aforementioned requirements.

### 2.4. Special Instructions on Laser Source

Due to the excellent frequency stability of He-Ne lasers, they are widely used in laser interferometry and even as the first-choice light source. In the beginning, a self-made double-longitudinal-mode thermal-frequency-stabilized He-Ne laser is used as the light source. The use of He-Ne light sources is not a problem in other applications, but some issues encountered in this high-bandwidth heterodyne laser interferometer require additional attention.

**Figure 6:**
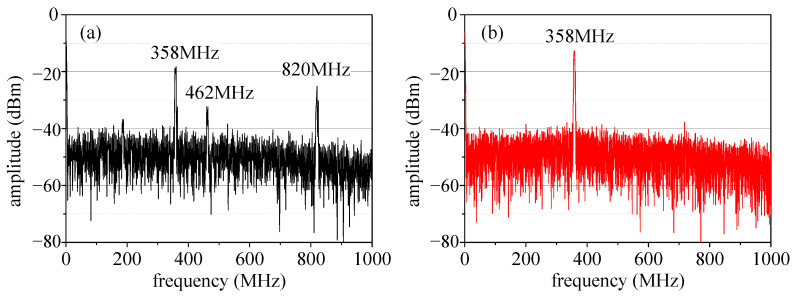
Spectrum of the interference signal when using different lasers. (**a**) He-Ne laser, (**b**) semiconductor laser.

When the target is stationary, by testing the spectrum of the interference signal output using the photodetector, it is found that an unexpected frequency component appears in the interference signal, as shown in Figure 6a. The frequency components contained in the interference signal include 358 MHz, 462 MHz, and 820 MHz, of which 358 MHz is the AOM frequency shift and 820 MHz is the mode spacing of the He-Ne laser. The frequency component of 820 MHz appears in the interference signal because the output light of the He-Ne laser is mixed with other light with a frequency difference of 820 MHz, which is determined by the working principle and internal structure of the light source. After the splitting of the PBS and the frequency shift of AOM, the beams of multiple frequencies interfere with each other to form multiple frequency components in the Interference signal. The frequency component of 462 MHz in the interference signal is formed by the interference of light beams with frequencies of *f*_1_ + 358 MHz and *f*_1_ + 820 MHz.

Because the spectrum of the interference signal contains undesired frequency components, an appropriate filter must be selected to filter the signal. Therefore, the actual available optical bandwidth of the system will be less than 104 MHz (462 MHz–358 MHz). It can be seen that the spectral characteristics of the laser light source will affect the bandwidth of the laser heterodyne interference system.

When the light source is replaced with a semiconductor iodine absorption frequency-stabilized laser with a pure optical spectrum, the spectrum of the interference signal is shown in Figure 6b. It can be seen that the interference signal spectrum contains only a frequency of 358 MHz. Theoretically, the available optical bandwidth of a heterodyne interference system is 358 MHz, which is greatly improved compared with the He-Ne laser source. Therefore, it is necessary to pay extra attention to the spectral characteristics of the light source, which can easily be overlooked during design.

## 3. Preliminary HIFU Sound Pressure Measurement Results

**Figure 7:**
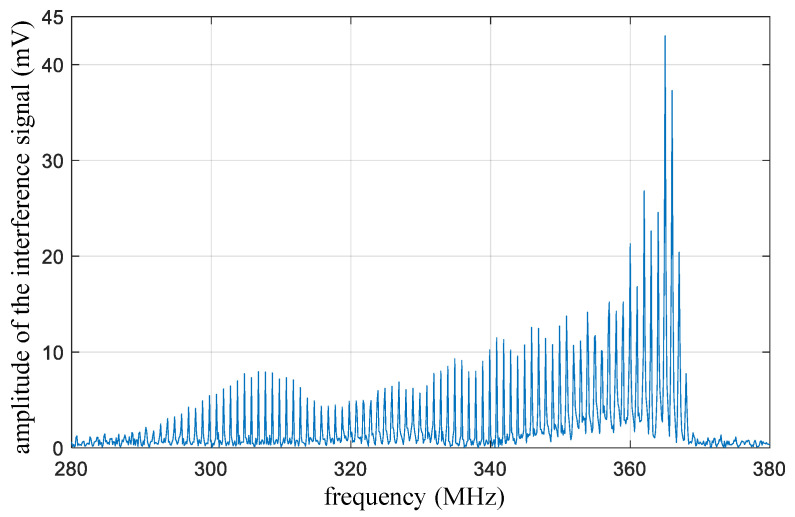
The spectrum diagram of the interference signal measured by the interference system.

The developed high-bandwidth laser heterodyne interferometer is used for an HIFU sound pressure measurement. A self-made HIFU transducer and a thin plastic reflective pellicle are placed in the water tank, and the interferometer measures the vibration of the pellicle through the optical window. The working frequency of the HIFU transducer is 1 MHz, which is close to the operating frequency of the HIFU treatment. The spectrum diagram of the interference signal measured at the focal point of the sound field at the maximum sound output power is shown in Figure 7. The interference signal exhibits an asymmetric distribution near the optical carrier frequency of 358 MHz, and the bandwidth of the interference signal reaches approximately 150 MHz.

**Figure 8:**
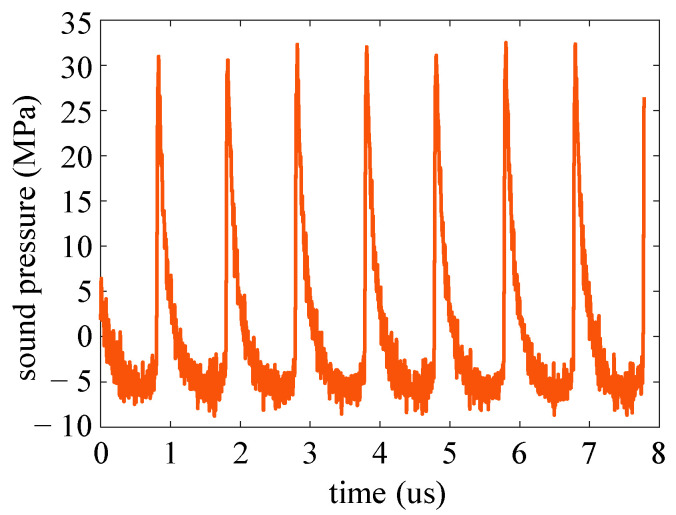
HIFU sound pressure curve measured by the interference system.

The corresponding HIFU sound pressure curve is calculated and shown in Figure 8, and the peak-to-peak value of the sound pressure is about 40 MPa. The maximum sound pressure measured by the interferometer is currently limited by the maximum output power of the HIFU transducer and the amplification ability of the power amplifier. After adding a higher power sound source, the interference system will have better performance.

## 4. Analysis of Measurement Uncertainty

The uncertainty components of the sound pressure measurement introduced by the interference system mainly include the frequency stability of the light source, the spatial averaging effect, a periodic nonlinear error in displacement measurement, the interference system bandwidth, and the photoelectric conversion process.

### 4.1. Frequency Stability of the Light Source

The light source used in this article is a semiconductor iodine-stabilized laser with a wavelength of 632.8 nm. Its relative frequency stability is better than 1 × 10^−7^, and the variation of the air refractive index is generally less than 1 × 10^−7^, which can be ignored compared to other uncertainty components.

### 4.2. Spatial Averaging Effect

The measurement light of the laser heterodyne interferometer is focused by a lens group, and the focal field diameter is about 10 μm. Therefore, it is not an ideal point receiver, which introduces the spatial averaging effect.

The correction of spatial average effects can be based on the method proposed by NPL [21]. The main method is to collect the sound pressure distribution at the focal point of the focusing transducer and then calculate the −6 dB beamwidth of each harmonic. Then the spatial average effect correction value at each frequency point can be expressed as follows:

(8) $$k_{sp}\left(j\right)=1+\frac{0.3}{\alpha_{j}^{2}-03}$$

where *α_j_* represents the effective radius of the −6 dB sound beam width at each frequency point. The diameter of the optical focal field is relatively small compared to the sound pressure focal field, so it can be considered that the uncertainty component introduced by the spatial averaging effect is less than 1%.

### 4.3. Periodic Nonlinear Error in Displacement Measurement

Firstly, by utilizing spatial separation optical paths, high-pass filtering of interference signals, and the Hilbert transform to obtain orthogonal signal equivalence, the generation of nonlinear errors can be greatly suppressed. In this system, the periodic nonlinear error of the displacement measurement can be expressed as follows:

(9) $$e_{x}\left(t\right)=a_{ex}cos\left[2\pi f_{c}t+\frac{4\pi nx(t)}{\lambda }\right],$$

where *a*_ex_ represents the amplitude of the nonlinear error. When the carrier frequency is 358 MHz and an oscilloscope with a sampling rate *F*_s_ = 20 GHz is used for interference signal acquisition, the average number of sampling points in each carrier cycle is about 56. Therefore, the maximum variation of the displacement nonlinear error between adjacent sampling points is about 0.11*a*_ex_. After the differential operation and sound pressure conversion, the maximum value of the sound pressure error can be expressed as follows:

(10) $$e_{p-max}={0.11a}_{ex}F_{s}\rho c,$$

When the nonlinear error in the displacement measurement is 0.1 nm, the uncertainty in the sound pressure measurement caused by it is about 0.3 MPa. When the sound pressure is 40 MPa, the measurement uncertainty caused by it is less than 1%.

### 4.4. Interference System Bandwidth

According to the analysis in this article, the uncertainty introduced by the system bandwidth is less than 2% when measuring a sound pressure below 100 MPa.

### 4.5. Photoelectric Conversion Process

The photoelectric conversion process includes photodetectors, cables, and oscilloscopes, mainly manifested as a non-flat amplitude response or a nonlinear phase response. After calibration [22], the uncertainty component caused by the photoelectric conversion process is less than 1%.

The components of uncertainty and the combined uncertainty are shown in Table 1. Within the designed sound pressure measurement range, the uncertainty introduced by the interference system is better than 5.3%.

## 5. Discussion

Due to the maximum output power of the HIFU sound source, the performance of the interferometer has not been fully tested here. However, we believe that the interferometer can meet the measurement requirements of higher HIFU sound pressures of up to 100 MPa in both the optical and electrical bandwidth. Next, we will actively obtain HIFU sound sources with a higher output power to test the performance of the interferometer. On this basis, we will conduct measurements and related metrology of the HIFU sound pressure at a level of 100 MPa based on this interferometer. In the process of achieving this goal, the high-bandwidth laser interference system is one of the key issues and a necessary condition for completing the measurements. In the future, we still need to solve many problems, such as the impact of the acoustic impulse flow and the issue of film tracking under high sound pressures.

## 6. Conclusions

In conclusion, a high-bandwidth laser heterodyne interference system for an HIFU sound pressure measurement was developed and tested in this paper. An AOM was used to obtain optical carrier frequencies up to 358 MHz. The bandwidth of the electrical part is higher than 1.5 GHz. The influence of the spectral purity of different types of laser sources on the bandwidth of the interference system was analyzed and tested. The preliminary HIFU sound pressure test results show that the interference system has good performance. Next, the output power of the HIFU sound source will be increased to achieve higher HIFU sound pressure measurements. This work can significantly improve the upper limit of HIFU sound pressure measurements based on laser interferometry and provide a reference and basis for the design and construction of laser heterodyne interferometry systems for HIFU sound pressure measurements. It is of great significance to improve the metrology system for therapeutic ultrasound.

## Figures and Tables

**Table 1:** Uncertainty components and combined uncertainty.

Uncertainty Component	Value
Frequency stability of the light source	≈0
Spatial averaging effect	1%
Periodic nonlinear error in displacement measurement	1%
Interference system bandwidth	2%
Photoelectric conversion process	1%
combined uncertainty	2.64%
expanded uncertainty (*k* = 2)	5.3%

## Data Availability

The data that support the findings of this study are available upon reasonable request from the authors.
